# Application of flexible pin for planetary gear set of wind turbine gearbox

**DOI:** 10.1038/s41598-022-05828-1

**Published:** 2022-02-02

**Authors:** Ho-Gil Yoo, Woo-Jin Chung, Beom-Soo Kim, Young-Jun Park, Su-Chul Kim, Geun-Ho Lee

**Affiliations:** 1 Department of Biosystems Engineering, Seoul National University, 1 Gwanak-ro, Gwanak-gu, Seoul, 08826 Republic of Korea; 2 Convergence Major in Global Smart Farm, College of Agriculture and Life Sciences, Seoul National University, 1 Gwanak-ro, Gwanak-gu, Seoul, 08826 Republic of Korea; 3 Research Institute of Agriculture and Life Sciences, Seoul National University, 1 Gwanak-ro, Gwanak-gu, Seoul, 08826 Republic of Korea; 4 Department of Smart Industrial Machinery, Korea Institute of Machinery and Materials, 156 Gajeongbuk-ro, Yuseong-gu, Daejeon, 34103 Republic of Korea

**Keywords:** Wind energy, Mechanical engineering

## Abstract

Wind turbines are eco-friendly energy sources that generate electricity from wind power. Among their various components, gearboxes constitute the most critical loss owing to their longest downtime. To guarantee their durability, a flexible pin was designed based on the original straddle-mounted pin for enhanced tooth load sharing and distribution in the planetary gear set (PGS) of a wind turbine gearbox (WTGB). The improved durability was evaluated by calculating the mesh load factor and face load factor for contact stress and comparing these values with those of the original straddle-mounted pin. The mesh load factor decreased from 1.37 to 1.08, whereas the maximum face load factor decreased slightly, moderating the overall safety factor variation. Furthermore, the structure of the proposed flexible pin model was analyzed and verified that no static failure or interference occurred. Additionally, microgeometry optimization was applied to improve the load distribution. Therefore, it was verified that a flexible pin applied to a single helical-geared PGS, thus far considered impossible, enhances the durability of WTGBs by improving the load sharing and distribution of a PGS. Consequently, the possibility of designing single helical-geared planetary gearboxes with flexible pins to take advantages of both helical gears and flexible pins was shown analytically.

## Introduction

Numerous countries have proposed environmental regulations to replace fossil fuels with environmentally friendly and renewable energy resources^1,2^. Among these, wind energy constitutes the largest proportion of renewable energy sources in the United States^3^, and wind turbines are used to effectively convert the kinetic energy of wind into electrical energy. They comprise rotating blades mounted on a rotor transmitting power to the main shaft, a generator for converting mechanical energy into electrical energy, a gearbox that switches the rotating speed of the main shaft to the rated speed of the generator, and surrounding structures that include the tower and rotor yaw mechanism. Typically, the blades and rotor are rotated by the wind at a low speed of up to 10–20 rpm, transmitting a large torque. However, induction generators produce electricity with adequate power at 1000–2000 rpm. Therefore, most wind turbines require a gearbox to increase the rotor speed, and a planetary gear set (PGS) is preferred owing to its high-power density and concentric input and output shafts. The high input torque of the blades engages the largest load at the first PGS^4^. Moreover, the gearbox is a complex component of a wind turbine, and as such has the longest downtime, constituting a critical loss^5^. Hence, improvement of the durability of wind turbine gearboxes (WTGBs) has been explored in numerous studies.

Typically, a WTGB comprises several stages of the PGS and parallel-axis gear pairs. The load sharing capacity of the former ensures a higher power density in the gear set than that of the latter. In other words, the PGS allows the designer to increase the gear ratio and power capacity of the gearbox within a limited space. However, as the first stage of the PGS must endure a high load, manufacturing and assembly errors can lead to PGS failure if the load is concentrated on a particular planet under uneven load sharing conditions. Additionally, it can result in misalignment and transmission error, which is the primary cause of gearbox vibrations and noise^6–10^. Therefore, several researchers have analytically measured and predicted the load sharing performance based on the carrier pinhole position error (PPE) and non-torque load^11–15^. Singh reported that a tangential PPE exhibits a more significant effect on the load sharing performance of PGS than a radial PPE^15^.

Increasing the number of planet gears effectively increases the power density of a PGS. However, Ligata et al. reported that a four-planet PGS may have a lower load sharing performance than a three-planet PGS, concluding that a higher number of planets can increase the risk of deteriorated load sharing performance^7^. Furthermore, the utilization of elastic components, such as floating members and flexible pins in a PGS, has been investigated to address the uneven load sharing. Typically, floating components are utilized in a PGS to improve its load sharing performance, as they eliminate the constraint of bearing and enable free movement of components in the radial direction^15,16^. However, Chung et al. reported the improvement in load sharing performance is limited when non-torque load and carrier PPE are considered^17^.

Hicks pioneered the concept of flexible pins in the 1960s to improve the load sharing performance of PGSs^18,19^. Approximately 40 years later, Fox and Jallat patented a revised flexible pin model exhibiting a better load sharing performance than that of Hicks, particularly under high loads due to the self-aligning effect. Additionally, they reported that spur gears are preferred over helical gears in designing flexible pins because even a small helix angle can generate thrust forces causing overturning moments, which may not achieve optimal compliance^20,21^. Montestruc designed a tapered flexible pin model and compared the finite element analysis (FEA) results with those reported by Hicks and Fox and Jallat, emphasizing that the maximum stress and load sharing performance vary with the pin geometry^22^. Furthermore, Prueter et al. highlighted the effect of pin tilting error and eccentricity of components on the load distribution of the tooth face in a flexible pin system using FEA and experiments. Their study analytically proved that lead modifications can compensate for the edge contact triggered by the poor load distribution^23^. Zhu et al. designed a dynamic model of a WTGB with a flexible pin and conducted FE-based dynamic simulations using time-dependent pin stiffness and gear transmission error as system excitations^24,25^. Wang et al. applied a flexible pin to a double helical-geared PGS of a geared turbofan engine. They designed dynamic models of original solid pins and flexible pins based on the models of Hicks, Fox and Jallat, and Montestruc and analyzed their load sharing performances^26^.

Existing studies primarily focused on flexible pins in the design stage as they improve the load sharing performance of PGSs. However, Fox and Jallat reported that applying a flexible pin to a single helical-geared PGS can deteriorate the load distribution on the tooth surface owing to the axial component of the gear mesh force causing tilting moments and gear body misalignment^21^. Additionally, Montestruc reported that flexible pins can be used in spur-geared or double helical-geared PGSs as they can counterbalance the overturning forces despite the existence of helix angles^22^. Therefore, flexible pins have been predominantly used in spur-geared PGSs. As a result, the advantages of helical gears, such as improved load distribution based on a higher contact ratio, lower levels of noise and vibration, and large power have not been utilized by flexible pins.

The objective of this study was to modify the PGS of a WTGB to increase the power density and reduce the load on the planet gears. A flexible pin was designed and applied to the PGS for improved load sharing performance and load distribution on each tooth. The effects of each design parameter of the pin on load sharing and distribution were analytically investigated to determine its optimal geometry. The flexible pin was applied to a single helical-geared PGS rather than a spur-geared PGS to improve both load sharing and distribution, and was designed to prevent the load distribution deterioration due to the overturning force. The study results indicate that the designed flexible pins can be successfully applied in a single helical-geared PGS, which appeared impractical thus far. Furthermore, the feasibility of the flexible pin was evaluated by comparing its strength with that of the original straddle-mounted planet pin. Finally, the tooth load distribution was improved via tooth lead modification. Thereby, the advantages of both helical gears and flexible pins could be exploited and the applicability of the proposed flexible pin was demonstrated to a single helical-geared PGS.

## Methods

### Performance quantification parameters

Two factors, namely the mesh load factor ($K_{\gamma }$) and face load factor for contact stress ($K_{H\beta }$), were calculated to evaluate the load sharing and tooth load distribution of a gear pair using the gear rating standard (ISO 6336)^27^. The factor $K_{\gamma }$ considers the uneven load applied to each path in a multipath transmission; in the case of PGS, it is the ratio of maximum to average torque. Alternatively, it can be calculated according to ANSI/AGMA 6123-B06^28^ as

1 $$ K_{\gamma } = \frac{{T_{branch} N_{CP} }}{{T_{nom} }}, $$

where $$T_{branch} $$ denotes the torque in the branch with the heaviest load (Nm),$$ N_{CP }$$ represents the number of planet gears, and $$T_{nom} $$ indicates the total nominal torque (Nm).

Ideally, the value of $K_{\gamma }$ is one when all planet gears share the load evenly. However, due to the existence of PPE, assembly error, and elastic deformation in a real scenario, *K*_*y*_ becomes larger than unity when the load does not split evenly.

The factor $K_{H\beta }$ is the ratio of the maximum load to the average load per unit face width, which considers the effect of non-uniform load distribution on the gear face width based on the surface contact stress. It results from manufacturing errors, thermal deformation, and elastic deformations of gears, bearings, shafts, and housing, and is calculated by:

2 $$ K_{H\beta } = \frac{{W_{max} }}{{W_{m} }} = \frac{{\left( {F/b} \right)_{max} }}{{F_{m} /b}}, $$

where $W_{max}$ denotes the maximum load per unit face width (N/mm), $W_{m}$ represents the mean load per unit face width (N/mm), $F_{m}$ indicates the mean transverse tangential load at the reference circle relevant to mesh calculations (N), and $b$ denotes the gear face width (mm).

### Requirements of WTGB and analysis methods

As $K_{\gamma }$ and $K_{H\beta }$ originate in gear rating standards, they are typically applied to evaluate the safety factors of gears. The ISO 6336 standard presents all procedures and formulae for calculating the gear contact safety factor ($S_{H}$) and bending safety factor ($S_{F}$) based on $K_{\gamma }$ and $K_{H\beta }$^27^. The Germanischer Lloyd (GL) guideline, widely known as a guideline for wind turbine design, requires that the load capacity of gears be verified for all engagement situations in the main gearbox of a wind turbine. The minimum values of safety factors ($S_{H}$ and $S_{F}$) required for fatigue analysis are 1.2 and 1.5, respectively^29^, which are calculated based on ISO 6336 standards. The gear is considered to exhibit an insufficient load capacity if these values are lower than those set by the GL guideline. In this study, the gear material was 18CrNiMo7-6 steel with an allowable contact stress ($\sigma_{{H{\text{lim}}}}$) and bending stress ($\sigma_{{F{\text{lim}}}}$) of 1560 MPa and 450 MPa, respectively. The value of $K_{H\beta }$ was calculated based on the FE method to reflect the elastic deformations of the gearbox components. All gearbox analyses, including the calculation of $K_{H\beta }$, $K_{\gamma }$, and the safety factors, were performed using Romax Nexus (Romax Technology Ltd., Ver. R20, 2020), a commercial software package developed to design and analyze gearboxes^30^. Finally, the stress and deflection levels of the original and flexible pins were compared and using an FE structural analysis using the OptiStruct solver of HyperWorks (Altair Engineering Inc., Ver. 2020.1, 2020), which is a commercial software package for linear and non-linear analyses^31^.

## Original straddle-mounted WTGB modeling and analysis results

**Figure 1:**
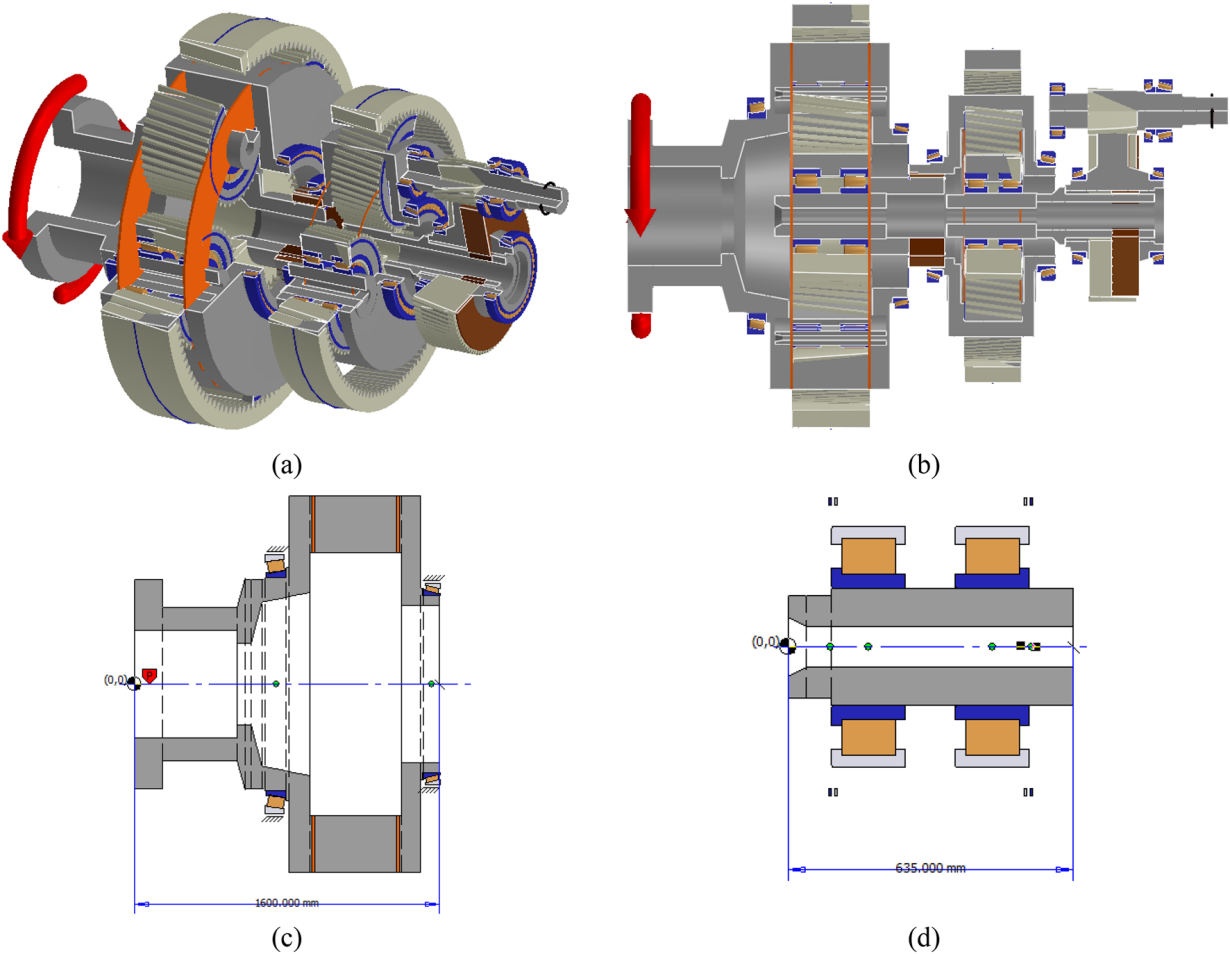
Target gearbox: (**a**) simulation model isometric view, (**b**) simulation model cut-away side view, (**c**) first-stage carrier shaft section plan, and (**d**) planet pin assembly section plan.

In this study, the gearbox of a 4.2 MW wind turbine with both two-stage and single-stage helical PGSs was the base model. The first and second stages of the PGS comprise five and three planets, respectively. Each planet gear is supported by two cylindrical roller bearings, and the ring gear is attached to the gearbox housing. The specifications of the target WTGB include a rated power of 4.2 MW, rotational speed of 6.3–12.2 rpm, operational temperature range of − 15 to 20 °C, survival temperature range of − 20 to 50 °C, and required lifetime of 20 years. Figure 1a,b depict the gearbox simulation model created using Romax Nexus. A flexible pin for the first stage of the PGS (Fig. 1c,d) was designed and the analytical results of the original and flexible pin models were compared. The major specifications of the first stage PGS is summarized in Table 1.

**Table 1:** The main specifications of the first PGS.

Items	Sun gear	Planet gear	Ring gear
Normal module (mm)	16		
Number of teeth	46	44	134
Face width (mm)	445	435	445
Normal pressure angle (°)	22.5		
Helix angle (°)	7		

A single-rated load case was determined based on the load duration distribution data. The rated input torque and rotating speed were set to 3,446 kNm and 10.9 rpm, respectively, corresponding to a power of 3.9 MW. The duration of the load case was set to 20 years, which is the required gearbox lifetime.

The occurrence of PPE prevents the placement of planet gears in ideal positions owing to the carrier manufacturing error, which leads to unintended pinhole positioning. Therefore, the planet gears do not mesh simultaneously and load sharing becomes uneven when one planet gear meshes relatively early. As PPE can be eliminated completely only when all elements of PGS are manufactured, machined, and assembled perfectly, this error must be considered during PGS design and analysis. In this study, a PPE of 0.0105° ($\theta_{{{\text{PPE}}}}$) about the center of the PGS was applied to the model based on the manufacturing tolerance of the target PGS.

**Figure 2:**
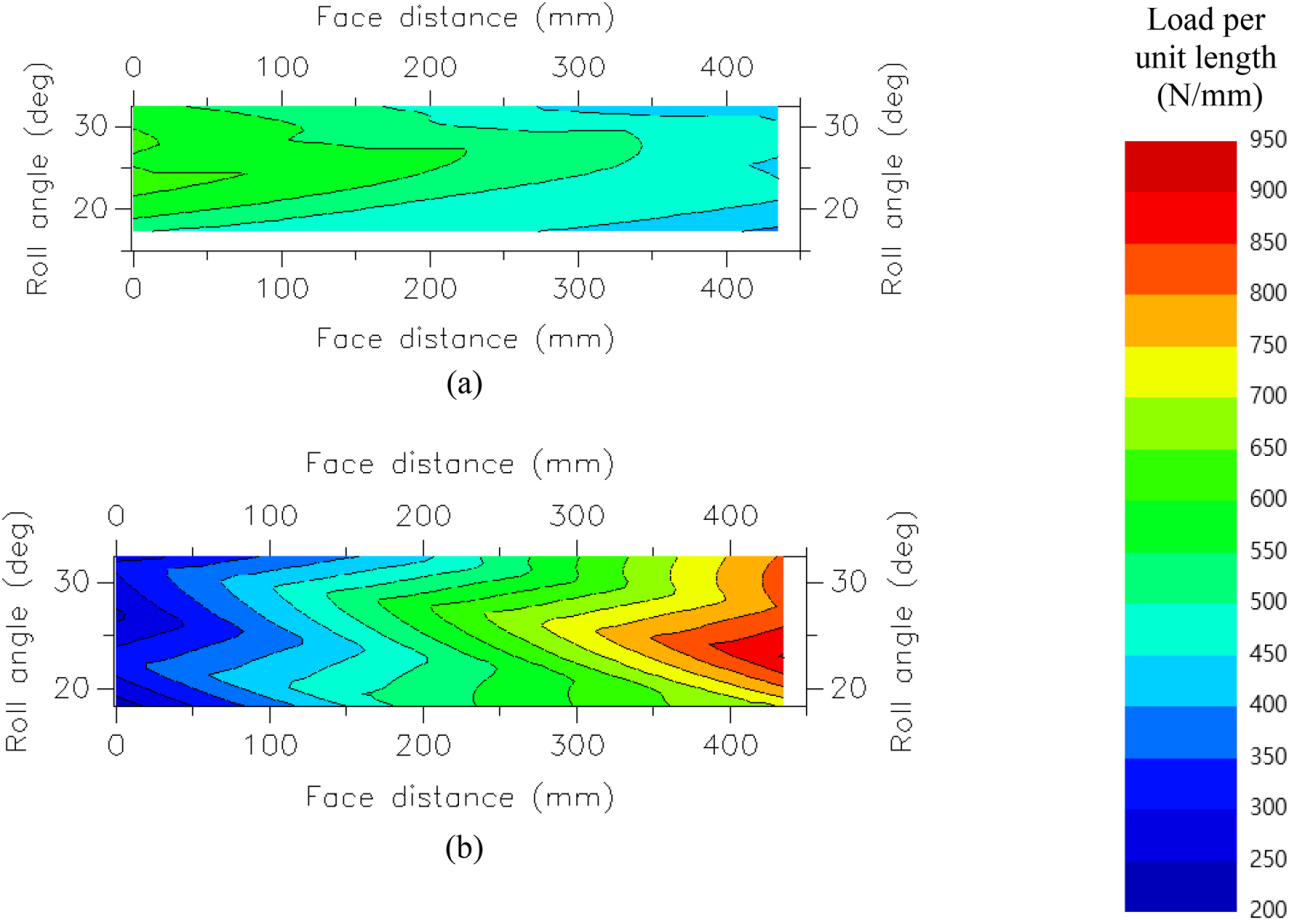
Contact patterns of the gear mesh of planet gear 2 in the original model: (**a**) ring–planet gear mesh (right flank), and (**b**) sun–planet gear mesh (left flank).

Table 2 presents the values of contact and bending safety factors, $K_{H\beta }$ and $K_{\gamma }$, respectively, of the first stage of the PGS in the original model. All meshes satisfied the required minimum safety factors (1.2 and 1.5), thus assuring the specified gearbox lifetime. However, the load distribution of the ring–planet mesh was poorer than that of the sun–planet mesh, resulting in a large $K_{H\beta }$. This can be graphically verified based on the contact patterns of each gear mesh (Fig. 2). For planet gear 2, which exhibits the largest $K_{H\beta }$ (1.49), the gaps between the maximum and minimum loads per unit length were 224 N/mm and 680 N/mm for the ring–planet and sun–planet meshes, respectively. This implies that the load was concentrated more severely at the edge of the latter, as illustrated in Fig. 2. The value of $K_{\gamma }$ was 1.37, which implies a non-uniform load distribution. Indeed, the torque was split unevenly between the gears, with values of 242.04, 124.98, 195.03, 195.42, and 125.56 KN⋅m at planets 1, 2, 3, 4, and 5, respectively. This equates to an average torque of 176.1 kN⋅m.

**Table 2:** Analytical results of the original model.

Gear	Contact safety factor		Bending safety factor		$K_{H\beta }$		$K_{\gamma }$
	Left flank	Right flank	Left flank	Right flank	Ring–planet	Sun–planet	
Ring gear	–	3.09	–	3.79	–	–	1.37
Planet gear							
1	1.65	3.14	1.86	2.25	1.08	1.30	
2	2.13	4.04	3.15	3.74	1.12	1.49	
3	1.91	3.44	2.48	2.71	1.08	1.20	
4	1.92	3.45	2.51	2.72	1.07	1.18	
5	2.19	4.09	3.32	3.81	1.08	1.38	
Sun gear	1.65	–	2.72	–	–	–

**Figure 3:**
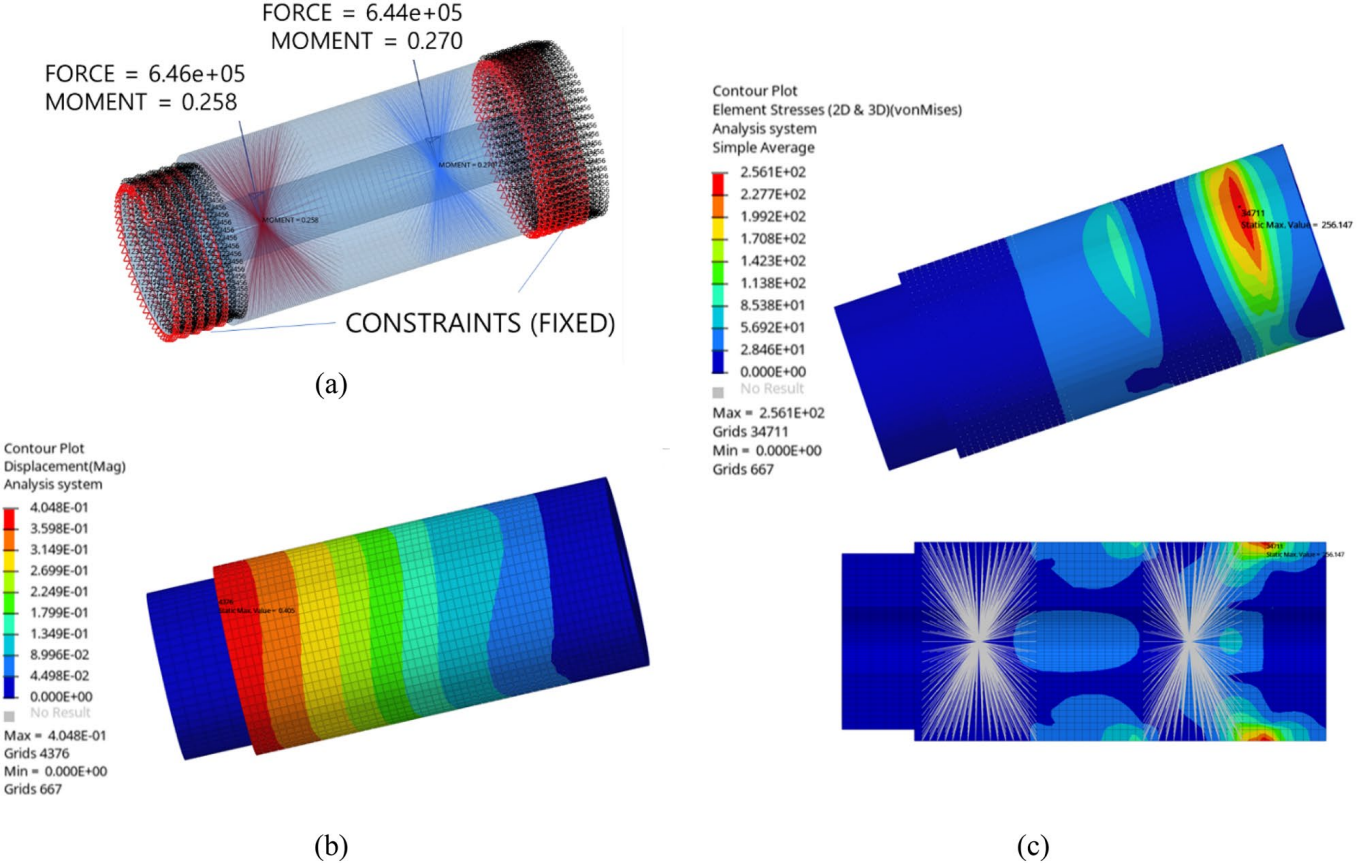
Finite element analysis and results of the original model: (**a**) boundary conditions and constraints (in kN and Nm), (**b**) displacement (in mm), and (**c**) von Mises stress (in MPa).

Finally, an FE structural analysis was performed to determine the pin stress and deformation levels. Figure 3 depicts the boundary conditions and results of FE structural analysis of the straddle-mounted pin model. The pin, which is mounted on the carrier in practice, was fixed to the ground in the simulation model. Both sets of bearing mounting surface nodes were bonded to the RBE2 component, and the 6-degree-of-freedom bearing force and moment from the quasi-static results of Romax Nexus were applied to each RBE2 component. The number of nodes and elements in the FE model were 39,902 and 35,102, respectively. The material properties used for the structural analysis included a Young’s modulus of 207 GPa, density of 7800 kg/m^3^, Poisson’s ratio of 0.29, yield strength of 380 MPa, and ultimate strength of 600 MPa. The maximum pin displacement was 0.4 mm, and the maximum von Mises stress was 256.1 MPa, which was less than the yield strength of the material.

## Flexible pin modeling and analytical results

**Figure 4:**
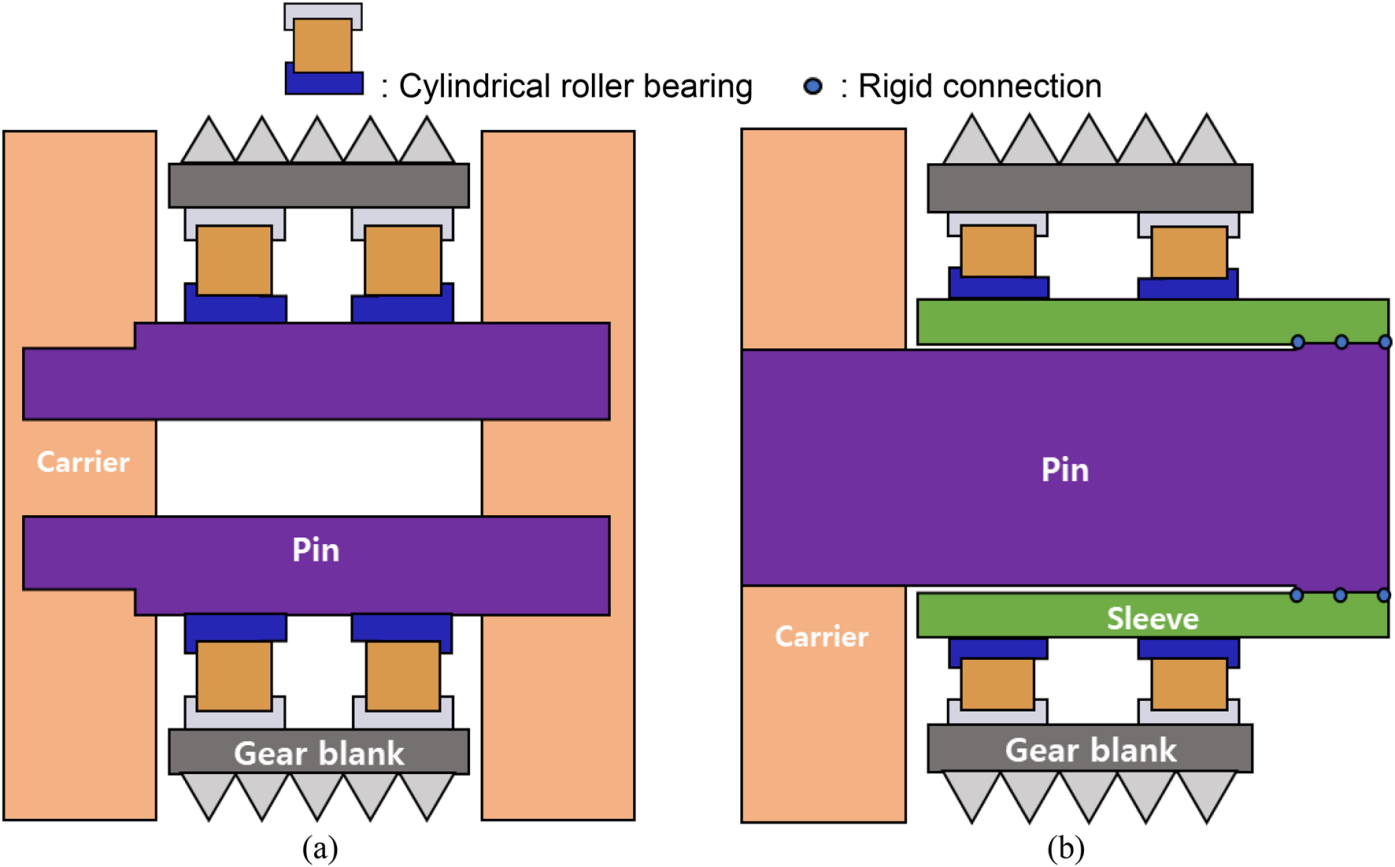
Schematics of the planet structure: (**a**) a conventional straddle-mounted pin, and (**b**) a flexible pin.

Figure 4a depicts the planet pins of the original PGS straddle-mounted on the carrier at both ends. In contrast, the flexible pin is cantilever-mounted on the carrier at one end, and a hollow shaft (sleeve) is fastened tightly using an interference fit at the other end; this is implemented in the simulation model using the rigid connection component (Fig. 4b). Unlike straddle-mounted pins, flexible pins deform to share the applied load evenly. For instance, if the pin of planet 1 is the most intensively loaded among five pins of a straddle-mounted PGS system, it deforms the most when the straddle-mounted pins are converted to flexible pins, thus dividing the excess load among the other pins. Consequently, the load sharing performance of the PGS system improves. This can be attributed to the gap between the pin and sleeve and relatively low pin deflection^32^. Therefore, no interference exists despite a narrow gap.

Although load sharing can be improved by applying a flexible pin, the face load distribution may deteriorate in the case of helical PGSs. Park et al. analytically verified that applying a flexible pin can increase $K_{H\beta }$^16^. Therefore, a flexible pin must be applied carefully to avoid increasing $K_{H\beta }$ as this will significantly affect the gear rating and life.

**Figure 5:**
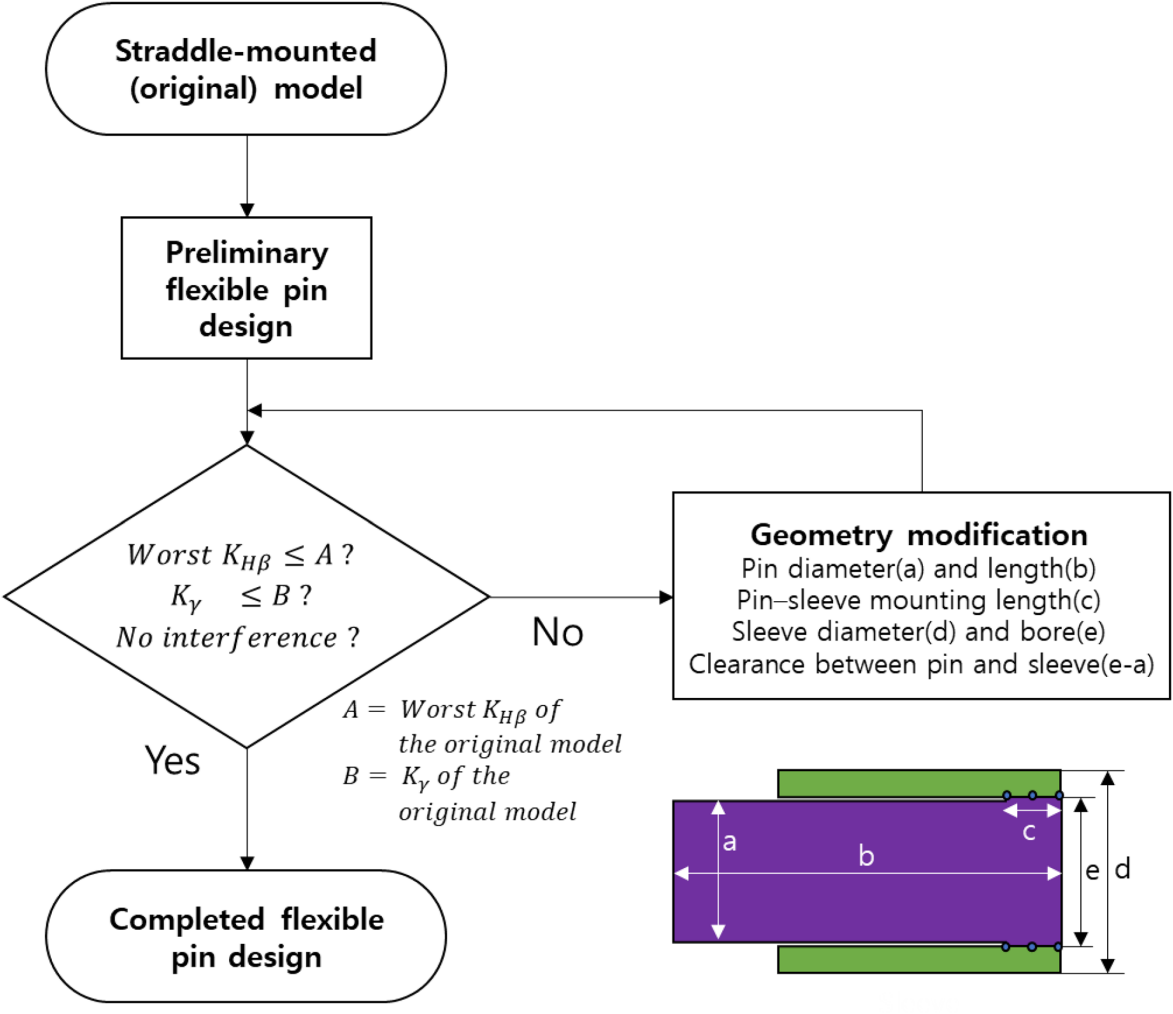
Design process of the flexible pin based on a straddle-mounted pin.

Figure 5 depicts the overall procedure used to design the preliminary flexible pin. After designing, evaluated $K_{H\beta }$, $K_{\gamma }$, and the existence of any interference were evaluated via simulations. In case of an unsatisfactory performance, the geometries of the pin, namely the pin diameter, pin length, mounting length between the pin and sleeve, sleeve bore, sleeve diameter, and clearance between the pin and sleeve, were modified until the criteria were satisfied. For instance, increasing the pin diameter improves the tooth load distribution but deteriorates load sharing. Therefore, in the case of excessively bad tooth load distribution, $K_{H\beta }$ can be moderately lowered by using a larger pin diameter despite the increase in $K_{\gamma }$. Additionally, other geometries and parameters, such as the mounting length between carrier and pin and bearing stiffness, collectively affect the overall performance. Therefore, the optimal geometries and parameters must be determined by trial-and-error.

A flexible pin for the first stage of the PGS was designed using the aforementioned process. The pin diameter was increased to compensate for the increased $K_{H\beta }$, and the roller bearing was reselected accordingly. A clearance of 35 mm was provided between the pin and sleeve to prevent interference caused by deflection.

Table 3 summarizes the analytical results of the first stage of the PGS under the rated input speed and torque considering the flexible pin model. Similar to the original model, every gear mesh satisfied the required minimum safety factors. The highest calculated $K_{H\beta }$ (1.49) decreased slightly to 1.45, moderating the variation of safety factors. Additionally, the value of $K_{\gamma }$ decreased by approximately 21% from 1.37 to 1.08. The reduced $K_{\gamma }$ and torque values of 181.48, 158.06, 170.78, 171.09, and 158.55 kN⋅m for planets 1, 2, 3, 4, and 5, respectively, with an average torque of 167.99 kN⋅m, indicate that the load sharing was considerably more even owing to the flexible pin.

**Table 3:** Analytical results of the proposed flexible pin model.

Gear	Contact safety factor		Bending safety factor		$K_{H\beta }$		$K_{\gamma }$
	Left flank	Right flank	Left flank	Right flank	Ring–planet	Sun–planet	
Ring gear	–	3.01	–	3.73	–	–	1.08
Planet gear							
1	1.80	3.07	2.25	2.22	1.44	1.37	
2	1.92	3.25	2.55	2.48	1.45	1.39	
3	1.95	3.18	2.62	2.37	1.41	1.24	
4	1.97	3.20	2.65	2.39	1.39	1.22	
5	1.99	3.28	2.73	2.52	1.42	1.29	
Sun gear	1.79	–	3.29	–	–	–	

**Figure 6:**
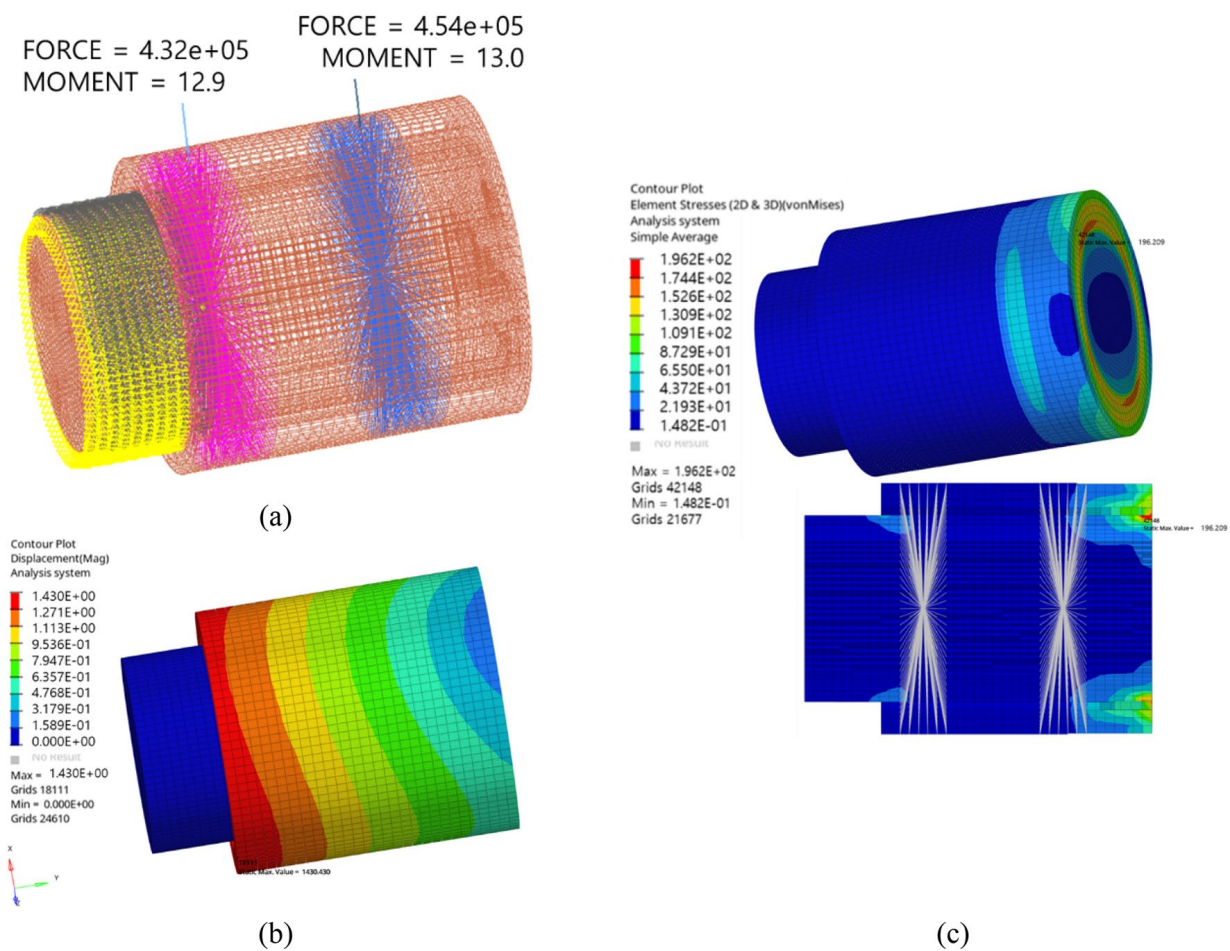
Finite element analysis and results of the flexible pin model: (**a**) boundary conditions and constraints (in kN and Nm), (**b**) displacement (in mm), and (**c**) von Mises stress (in MPa).

Figure 6 illustrates the boundary conditions and results of the FE structural analysis of the flexible pin model. The number of nodes and elements in the FE model were 57,456 and 50,885, respectively, and the material properties were identical to those of the original model. The carrier mounting nodes were fixed at the end of the pin, and the bearing loads calculated using Romax Nexus were applied to RBE2 components to fix the bearing mounting surface nodes. Consequently, the maximum pin displacement was 1.4 mm, which was not sufficiently large to exhibit any interference owing to the 35 mm clearance. The maximum von Mises stress was 196.2 MPa, which was less than the yield strength. Additionally, the stress level was lower than that of the original model.

### Microgeometry modifications for enhanced load distribution performance

The results verified that the flexible pin can effectively improve the load sharing performance. However, certain limitations in improving the load distribution exist when the original pin is under the edge contact condition. Typically, the load distribution of gear pairs can be improved by modifying the gear microgeometry. Cho et al. analytically verified that the load distribution of the high-speed stage of the helical gear set in a WTGB is affected by microgeometry modifications^33^, which, in this study, were used to improve the load distribution of the flexible pin model based on the tooth face width.

**Figure 7:**
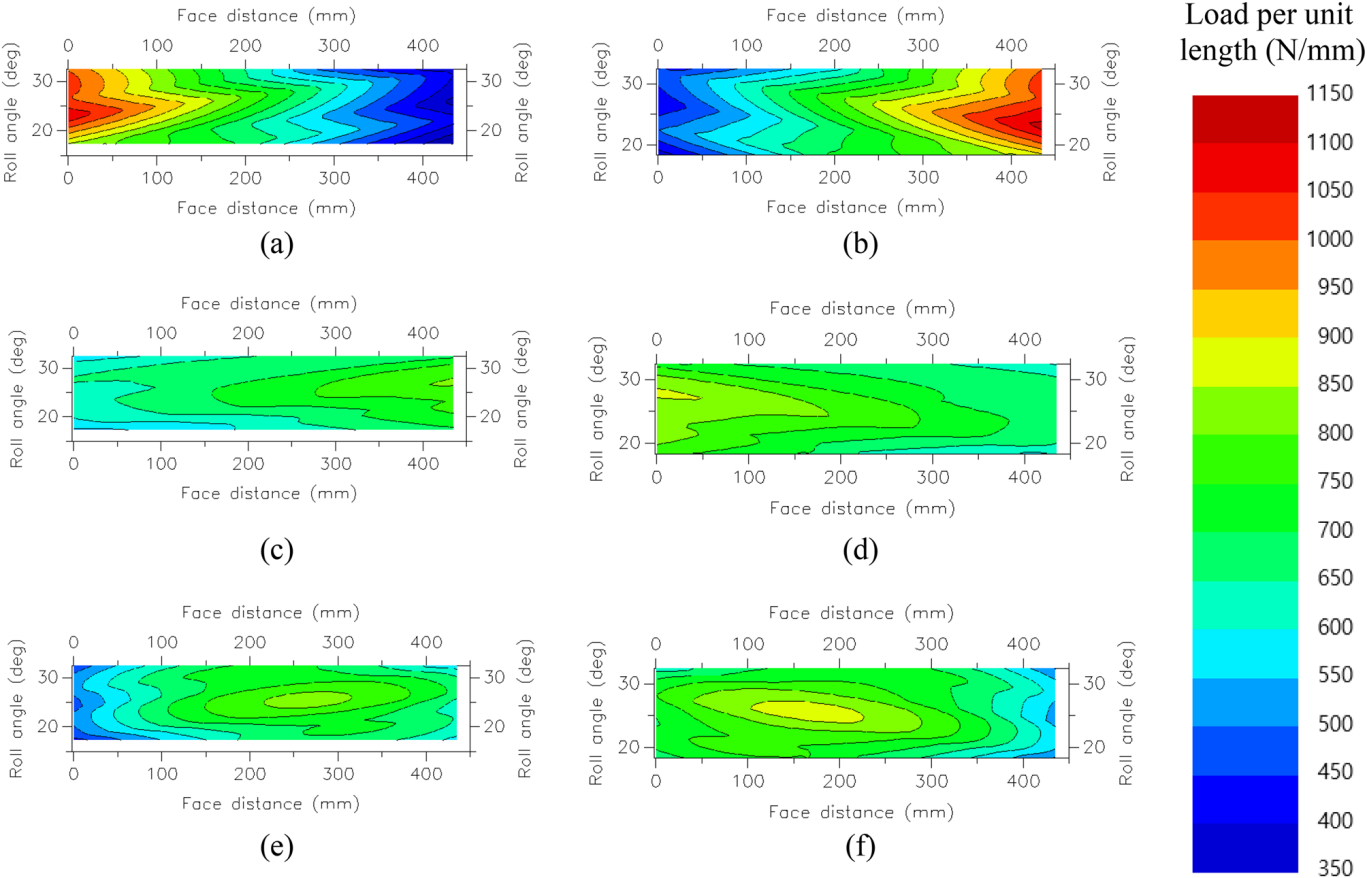
Contact patterns of the gear mesh of planet gear 2 in the flexible pin model with and without lead modifications: (**a**) ring–planet (right flank) without modification, (**b**) sun–planet (left flank) without modification, (**c**) ring–planet (right flank) with slope modification, (**d**) Sun–planet (left flank) with slope modification, (**e**) ring–planet (right flank) with slope modification and crowning, and (**f**) sun–planet (left flank) with slope modification and crowning.

Microgeometry modification, referred to as the tooth flank modification in ISO 21,771^34^, represents the intentional modification of specific parts of the involute tooth flank based on the primary geometry. For instance, profile and lead modifications signify modifications of involute-wise and face width-wise tooth profiles, respectively. If a gear pair is under the edge contact condition (Fig. 2), $K_{H\beta }$ can be adjusted to obtain a value close to unity with proper lead modification, which is classified into slope modification, in which the lead face is modified to reduce the thickness of the tooth on one end while retaining the original thickness on the other end, and crowning, in which the lead face is modified to reduce the thickness of the tooth on both ends while retaining the original thickness in the middle^34^. If the slope modification is applied to the edge-loaded part, the load is distributed on the entire tooth face, achieving an even load distribution. To minimize $K_{H\beta }$ of the flexible pin model, the optimal extent of slope modification was derived through a parametric study. It was determined that slope modifications 35 μm and 40 μm from the edge-loaded end of the left and right flanks, where planet gears mesh with the sun and ring gears, respectively, can ensure an optimal load distribution on each tooth flank. Figure 7a,b illustrate the unmodified analytical results, and Fig. 7c,d show those after slope modification. The range of load per unit length was decreased from 300–1000 N/mm to 500–850 N/mm at the right flank of the tooth and from 350–1150 N/mm to 550–950 N/mm at the left flank of the tooth, indicating that the load distribution improved significantly. However, as the load was not concentrated at the tooth center, unexpected edge loading may result in overload and fracture. This situation can be prevented via crowning, which ensures that the load remains concentrated at the center of the tooth by grinding each end. Therefore 5 μm of crowning was applied on the left and right flanks of the teeth on each planet gear, and the results are illustrated in Fig. 7e,f. Table 4 summarizes the analytical results. It was observed that the load distribution was centered owing to slope modification and crowning, thus preventing contact with the edge. Consequently, $K_{H\beta }$ decreased to approximately 1.1 on average, which was substantially smaller than that observed without microgeometry modification. However, the value of $K_{\gamma }$ did not change because load sharing in the model was unaffected by the microgeometry modification. Moreover, the safety factors simultaneously increased with decrease in $K_{H\beta }$.

**Table 4:** Analytical results of the flexible pin model with microgeometry modifications.

Gear	Contact safety factor		Bending safety factor		$K_{H\beta }$		$K_{\gamma }$
	Left flank	Right flank	Left flank	Right flank	Ring–planet	Sun–planet	
Ring gear	–	3.34	–	4.52	–	–	1.08
Planet gear							
1	2.01	3.43	2.73	2.70	1.11	1.11	
2	2.12	3.60	3.05	2.97	1.13	1.13	
3	2.04	3.50	2.82	2.80	1.13	1.14	
4	2.03	3.49	2.80	2.79	1.13	1.15	
5	2.11	3.58	3.02	2.95	1.14	1.14	
Sun gear	1.95	–	3.96	–	–	–	

## Discussion

At the design phase, the flexible pin was designed to enhance the load sharing performance at the rated load case and a specific PPE value, which is the maximum manufacturing tolerance at a single pin hole. Even if the load sharing performance is improved at this condition, the effect of flexible pin is dependent on the extent of load or PPE. To investigate the robustness of load sharing effect of flexible pin against the various load levels and types of PPE, additional analysis has been conducted.

**Figure 8:**
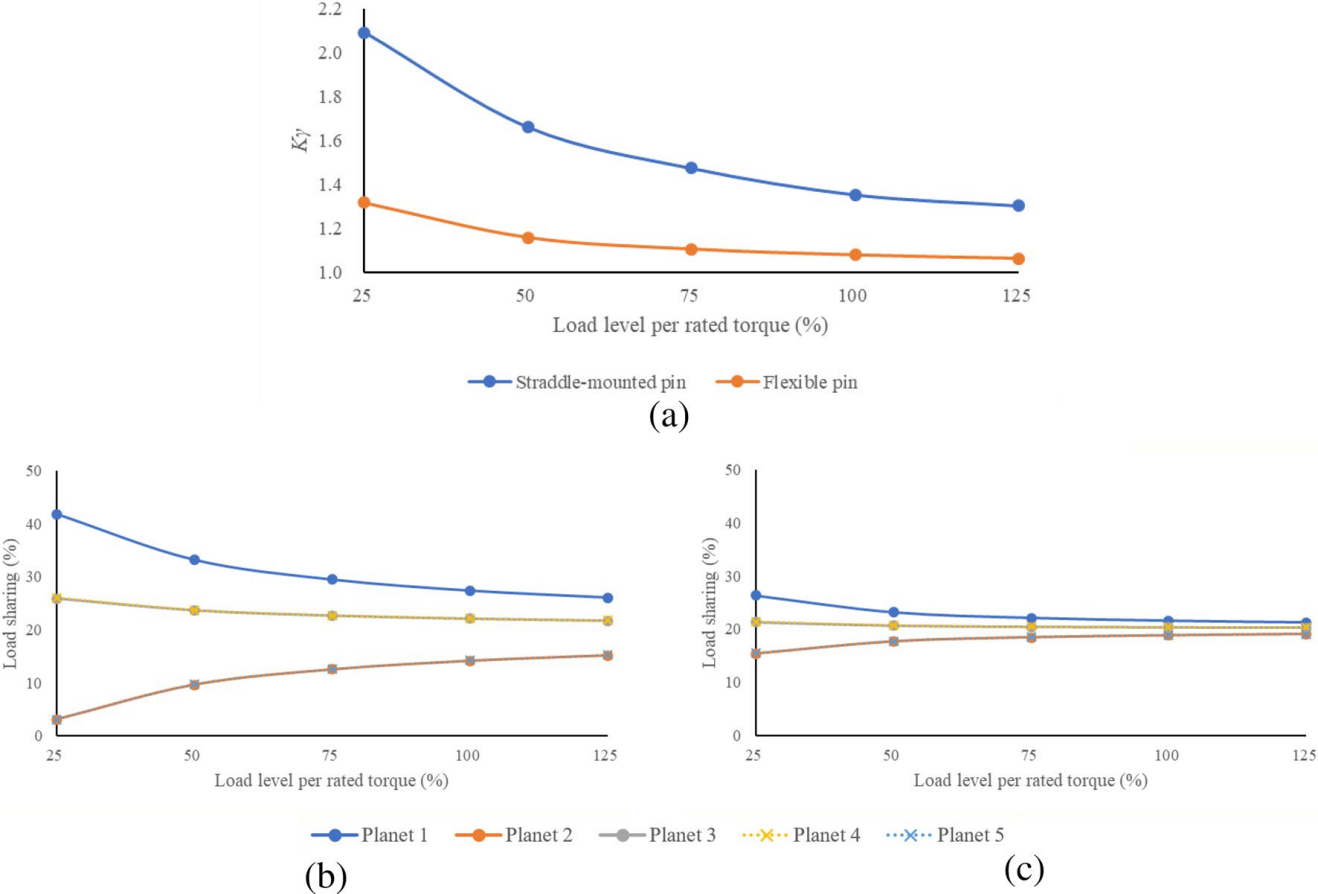
Load sharing performance of straddle-mounted pin and flexible pin under various load level: (**a**) load sharing ratio $K_{\gamma }$, (**b**) load shared by each planet (straddle-mounted pin), and (**c**) load shared by each planet (flexible pin).

At first, to evaluate the effect of the flexible pin at the various load levels, $K_{\gamma }$ of PGS with straddle-mounted pin and flexible pin under 25, 50, 75, 100 and 125% of rated torque (3446 kNm) were calculated and compared in Fig. 8. Figure 8a shows that $K_{\gamma }$ is lower at every load case. Also, Fig. 8b,c indicate that the planet gears of PGS with flexible pin shares the load more equally than the original model, even when the torque is lightly engaged. Ideally, 20% of load should be shared at each planet. However, planet 1, where the PPE was applied, of straddle-mounted pin model holds 42% of total load and planet 2 and 5 hold 3% respectively, whereas planet 1 of flexible pin model holds 26% and planet 2 and 5 hold 15% of total load at the 25% of the rated torque. Likewise, the load sharing performance of flexible pin model at the excessive load (125% of the rated torque) is superior to the original model. Therefore, the load sharing performance would be better when flexible pin is applied regardless of the extent of load.

**Figure 9:**
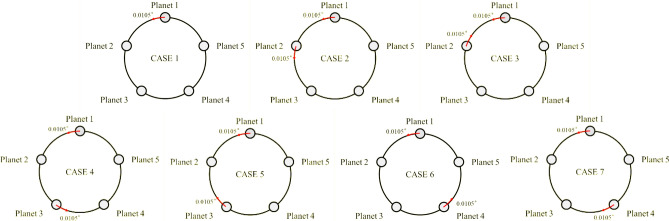
Various PPE analysis cases to ensure the robustness of load sharing effect of flexible pin.

Secondly, to ensure the load sharing performance of flexible pin, $K_{\gamma }$ under 7 PPE cases (Fig. 9) had been calculated and the results were summarized in Table 5. Case 1 is the reference PPE applied up to the above analysis and the other PPE cases were considered here additionally. As shown in Table 5, $K_{\gamma }$ of the flexible pin model was lower than the original model in every PPE case. Case 3 turned out to be the worst case for both models, indicating $K_{\gamma }$ of 1.66 in the original model and 1.14 in flexible pin model. Case 4 & 6 and Case 5 & 7 had equivalent effect, respectively. Additionally, the extreme amount of PPE (0.0315°) was applied for reference case (Case 1) and worst case (Case 3) and reported as Case 1a and 3a in Table 5. Case 1a and 3a showed that $K_{\gamma }$ of flexible pin is significantly lower than the original model (Case 1a: 2.10 & 1.26, Case 3a: 2.22 & 1.43). Therefore, the robustness of effect of flexible pin against the various PPE cases and the extent of PPE has been ensured.

**Table 5:** $K_{\gamma }$ evaluated under the various PPE cases.

PPE case	$K_{\gamma }$	
	Straddle–mounted pin	Flexible pin
1	1.34	1.08
2	1.21	1.03
3	1.66	1.14
4	1.47	1.10
5	1.39	1.07
6	1.47	1.10
7	1.39	1.07
1a	2.10	1.26
3a	2.22	1.43

In this study, a flexible pin was designed to improve the load sharing performance of the WTGB, and microgeometry modifications were applied to determine the load distribution on the gear flanks. Compared with the original model, $K_{\gamma }$ and $K_{H\beta }$ decreased to approximately 1.1, implying that load sharing performance and distribution improved considerably.

However, directly applying a flexible pin can increase the value of $K_{H\beta }$ when the pin diameters of the original straddle-mounted pin model and flexible pin model are identical, despite the decrease in $K_{\gamma }$. Hence, microgeometry modification was required to address this problem. The extent of lead slope modification was determined using a parametric study of the right and left flanks. As WTGB predominantly rotates in a single direction, the planet gear meshes with the sun and ring gears only at left and right flanks, respectively. This implies that $K_{H\beta }$ can be minimized by applying the slope modification determined from the parametric study.

In the meantime, the microgeometry modification was optimized for the rated torque (3446 kNm) only. In the other words, current microgeometry modification could be excessive for the partial torque (50% of the rated load, 1723 kNm) condition. To investigate the load distribution performance of microgeometry modified flexible pin model at the partial load condition, $K_{H\beta }$ of the flexible pin model with and without microgeometry modification under partial load were calculated and summarized in Table 6. While $K_{H\beta }$ under partial load were similar to the rated load when the microgeometry modification is not applied, $K_{H\beta }$ were increased under partial load condition when the microgeometry modification is applied. However, now that the extent of loaded torque was decreased, safety factors were rather increased despite deteriorated $K_{H\beta }$ (Table 7). Namely, even if the load distribution could be worse in the partial load condition than the rated load, the actual damage induced by poor load distribution of the partial load is not significant.

**Table 6:** $K_{H\beta }$ of the flexible pin model with and without microgeometry modification under the rated and partial load condition.

$K_{H\beta }$		No microgeometry modification		Microgeometry modification applied	
	Torque (%)	100%	50%	100%	50%
	(kNm)	3446	1723	3446	1723
Ring-planet mesh	1	1.44	1.45	1.11	1.33
	2	1.45	1.47	1.13	1.50
	3	1.41	1.39	1.13	1.44
	4	1.39	1.36	1.13	1.46
	5	1.42	1.41	1.14	1.55
Planet-sun mesh	1	1.37	1.38	1.11	1.30
	2	1.39	1.42	1.13	1.44
	3	1.24	1.20	1.14	1.44
	4	1.22	1.17	1.15	1.46
	5	1.29	1.37	1.14	1.48

**Table 7:** Safety factors of the flexible pin model with microgeometry modification under the rated and partial load condition.

	100% Torque (3446 kNm)				50% Torque (1723 kNm)			
	Contact safety factor		Bending safety factor		Contact safety factor		Bending safety factor	
	Left flank	Right flank	Left flank	Right flank	Left flank	Right flank	Left flank	Right flank
Ring gear	–	3.34	–	4.52	–	3.95	–	6.47
Planet gear								
1	2.01	3.43	2.73	2.70	2.46	4.08	4.17	3.88
2	2.12	3.60	3.05	2.97	2.64	4.31	4.84	4.37
3	2.04	3.50	2.82	2.80	2.47	4.13	4.24	4.00
4	2.03	3.49	2.80	2.79	2.45	4.10	4.19	3.96
5	2.11	3.58	3.02	2.95	2.60	4.26	4.73	4.28
Sun gear	1.95	–	3.96	–	2.38	–	6.00	–

Additionally, the pin geometry was optimized by modifying several parameters, which was challenging owing to the trade-off of $K_{H\beta }$ for each gear mesh associated with changes in certain parameters. For instance, increasing the pin diameter decreases $K_{H\beta }$ of both meshes; however, the diameter cannot be increased excessively because of the spatial constraints. Conversely, increasing the mounting lengths between the pin and carrier decreases and increases the $K_{H\beta }$ of the sun–planet and ring–planet meshes, respectively. Additionally, increasing the sleeve length has the opposite effect. Therefore, modifying the straddle-mounted pin into a flexible pin is difficult owing to spatial constraints. Moreover, the value of $K_{H\beta }$ can be decreased slightly or maintained at a similar level only when the optimal geometry is applied with a large pin diameter under no microgeometry modifications. To summarize, the pin geometry was optimized despite the challenges of increased $K_{H\beta }$ values when directly substituting the straddle-mounted pin with a flexible pin.

## Conclusions

A flexible pin was applied to a single helical-geared PGS of a WTGB to improve the load sharing performance and tooth load distribution. The effect and feasibility of the proposed flexible pin model has been analytically investigated. The load sharing performance improved significantly, and the load distribution performance remained similar to that of the original straddle-mounted pin model with a slightly decreased $K_{H\beta }$. Additionally, the flexible pin design ensured that all gear meshes satisfied the required safety factors, and the stress level in the pin was lower than the material yield strength. Furthermore, microgeometry modifications were applied to the proposed flexible pin model to compensate for the deterioration of $K_{H\beta }$ when applied to single helical-geared PGS. Thus, a method to improve load sharing and distribution through relatively simple design changes of the straddle-mounted single helical-geared PGS for WTGBs was presented. The analytical evaluation confirmed that a flexible pin can be applied to a single helical-geared PGS, which was previously considered impossible. In the future, the effect of the flexible pin must be verified by actual tests, and the WTGB can be designed to exploit the advantages of both helical gears and flexible pins.
